# Assessing Medication Adherence to Tadalafil 5 mg Once Daily in Erectile Dysfunction: A Cross‐Sectional Analysis

**DOI:** 10.1002/prp2.70129

**Published:** 2025-05-30

**Authors:** Emre Kandemir, Onur Kucuktopcu

**Affiliations:** ^1^ Faculty of Medicine, Department of Urology Karamanoglu Mehmetbey University Karaman Turkey; ^2^ Clinic of Urology Konya City Hospital Konya Turkey

**Keywords:** beliefs about medicines, erectile dysfunction, illness perception, medication adherence, medication beliefs, once daily, tadalafil

## Abstract

Our study aimed to examine medication adherence (MA) to tadalafil 5 mg once daily (OaD) in patients undergoing treatment for erectile dysfunction (ED) and to identify factors contributing to potential drug noncompliance. This cross‐sectional study included 233 patients diagnosed with ED. Sociodemographic and clinical data were recorded. MA was assessed using the Medication Adherence Report Scale (MARS). Additionally, the Brief Illness Perception Questionnaire (B‐IPQ), the Beliefs about Medicines Questionnaire (BMQ), and the International Index of Erectile Function (IIEF) were employed to evaluate patients' perceptions and beliefs regarding their condition and treatment. The influence of these factors on MA was thoroughly analyzed. High MA was reported in 136 (58.4%) of 233 patients. Factors, such as education level, monthly income, frequency of medical examinations, smoking habits, and a history of radical pelvic surgery, were found to influence MA (*p* < 0.05) significantly. Multivariate analysis identified monthly income and radical pelvic surgery history as statistically significant predictors of adherence (*p* ≤ 0.05). Additionally, adherence was significantly associated with IIEF scores, five items on the B‐IPQ, and the BMQ subscales, including specific concerns, necessity, and general harm (*p* < 0.05). Tadalafil OaD demonstrates acceptable rates of MA in the treatment of ED. Socioeconomic and clinical factors, patients' cognitive and sensory status, and perceptions regarding medications and healthcare providers significantly influence adherence. Physicians should exercise caution when prescribing tadalafil 5 mg OaD to patients with lower socioeconomic status, as they may be at higher risk for reduced MA.

## 1 Introduction

Erectile dysfunction (ED) is the inability to achieve or maintain an erection sufficient to achieve satisfactory sexual performance. ED affects the patient's and his partner's quality of life and psychosocial health [1]. The etiology of ED is multifactorial, often involving one or more vascular, neurogenic, hormonal, or psychogenic mechanisms. The initial approach to the treatment of ED involves lifestyle modifications and the correction of reversible risk factors identified through patient history. Phosphodiesterase type 5 (PDE‐5) inhibitors are used as an oral medical treatment option in patients who do not respond to this treatment [2]. Four different PDE‐5 inhibitors are approved by the European Medicines Agency [3]. These drugs can be used on demand (pro re nata [PRN]), three times a week, or once daily (OaD). However, studies have shown that using PDE‐5 inhibitors OaD significantly reduces the intracavernosal structural changes caused by chronic diseases [4]. Furthermore, it is more effective in promoting spontaneous sexual activity compared to PRN use [5]. It has been suggested that tadalafil OaD use should be maintained for a minimum of 12 weeks in patients with ED who also report lower urinary tract symptoms secondary to benign prostatic hyperplasia (BPH) [6]. However, the therapeutic effectiveness of these medications is primarily influenced by medication adherence (MA).

MA refers to the extent to which a patient accepts and consistently follows the prescribed drug regimen as recommended by healthcare professionals [7]. Properly using prescribed medications that adhere to the recommended dosage and schedule constitutes the most critical element of MA [8]. MA is particularly critical in chronic diseases due to the prolonged nature of pharmacotherapy. Numerous factors influence adherence, including treatment‐related side effects, duration of therapy, therapeutic efficacy, the patient's psychological state, medication costs, the impact of healthcare systems on drug pricing, and societal biases or stigmas associated with treatment [9]. Furthermore, evaluations of medication‐related hospitalizations reveal that nearly half of such cases are related to low MA [10]. As a result, MA is crucial for public health protection and the prevention of economic losses. However, no gold standard exists for monitoring patient compliance with prescribed drug regimens. Commonly used methods include measuring drug concentrations or metabolites in blood, serum, or urine, analyzing prescription data from administrative databases, utilizing electronic medication monitors, and relying on self‐reported adherence through questionnaires, diaries, or patient reports. However, none of these methods are entirely practical for routine daily use.

Patients' perceptions of the efficacy, safety, and appropriateness of PDE‐5 inhibitor dosages can vary. A common misconception among cardiac patients is that abstaining from sexual activity is necessary to prevent adverse cardiovascular events, such as heart attack [11]. These factors collectively contribute to irregular medication use, thereby adversely impacting treatment outcomes. To the best of our knowledge, our study is the first in our country to assess MA in patients receiving tadalafil 5 mg OaD treatment. This study aimed to evaluate drug adherence among ED patients and identify nonadherence factors.

## 2 Materials and Methods

### 2.1 Study Design

Patients who were followed up with the complaint of ED in the urology outpatient clinic between August 2021 and May 2024 and who received or were receiving tadalafil 5 mg OaD treatment for at least 12 weeks in the last year were included in the study. Those under the age of 18, those with psychiatric or cognitive disorders, those receiving psychiatric treatment, those who are incapable of reading and understanding booklets or questionnaires, those who cannot complete the questionnaire after completing only a part of the questionnaire, and those with severe comorbidities who need a caregiver were excluded from the study. The purpose of the study was explained to all patients, and informed consent was obtained. Ethics committee approval was obtained. Patients were called for follow‐up every 3 months. Follow‐ups were carried out in the form of face‐to‐face meetings in the clinic. Phone numbers of the patients were recorded. They were informed by phone during the follow‐up periods and called for a face‐to‐face examination. The frequency of the medical examination was recorded. Patients who never came for their follow‐up were excluded from the study.

### 2.2 Data Collection and Questionnaires

Age, marital status, education level, monthly income, duration of ED, frequency of medical examination, chronic diseases, smoking, and radical pelvic surgery history of the patients were recorded. Medication Adherence Report Scale (MARS) is a self‐reported five‐question questionnaire [12]. With these questions, the frequency of the patient's tendencies, such as forgetting to take medication, avoiding medication, changing the drug dose, or skipping the dose, is scored. Higher scores are obtained in patients who pay attention to the dose and order of the drug. Scoring ranging from 1 (always) to 5 (never) is used on this scale. Patients with scores of 20 and above are considered to have high MA. This study used the Turkish version of MARS, previously validated for measuring treatment adherence in all chronic diseases, similar to the original version [13]. In our study, the MARS scores of the patients were obtained. The patients were divided into two groups. A score below 20 was considered low MA (Group 1). A score of 20 and above was considered high MA (Group 2).

The Brief Illness Perception Questionnaire (B‐IPQ) consists of eight questions to measure patients' cognitive and emotional perceptions of their illness [14, 15]. There are scales between 0 and 10 for each question. The maximum score in B‐IPQ is 80. If the patient gets a high score on this scale, it shows they have a more negative perception of the disease.

The Beliefs about Medicines Questionnaire (BMQ) is a scale consisting of 18 questions that investigate general and specific beliefs related to the medication used by the patient regularly [16]. BMQ‐specific is where the specific treatment required by the person's disease is questioned. It consists of five items (specific necessity) in which the necessity of drug treatment is questioned and five (specific concerns) in which side effects and concerns related to drug therapy are questioned. BMQ‐general consists of eight items in which the patient's thoughts and prejudices against all drugs are investigated. The section where the belief that physicians give excessive and unnecessary drug treatment is investigated (general overuse). The section where the belief that drugs are harmful is investigated (general harm). The scale of specific beliefs has five questions ranging from 5 to 25. Higher scores in the Specific‐Necessity Subscale show that the patient believes more in the contribution of drugs to treating the disease. Higher scores in the Specific Concerns Subscale show higher concern that regular drug use will have adverse effects. Higher scores in the general overuse show that there is more thought that physicians are prescribing excess or unnecessary medication. Higher scores in the general harm show a stronger belief that drug use is harmful. The Turkish version of BMQ was used in our study [17].

There are many scales used in the evaluation of ED. The International Index of Erectile Function (IIEF) is one of the most widely used scales [18]. The version of this questionnaire adapted to our language has been commonly used in our country since 2002 [19]. Six questions were identified on the scale to assess the quality of erectile function. Each question can be scored between 0 and 5. The IIEF scores of the patients before receiving tadalafil 5 mg OaD treatment were recorded. According to these scores, the patients were grouped according to the scale mentioned above.

In our study, the MARS scores of the patients were obtained. The patients were divided into two groups (low MA: Group 1 and high MA: Group 2). Clinical and demographic data, B‐IPQ scores, BMQ scores, and IIEF scores of the patients were compared between the two groups.

### 2.3 Statistical Analyses

Data were analyzed using the Statistical Package for Social Studies (IBM Corp. Released 2018. IBM SPSS Statistics for Windows, Version 23.0, Armonk, New York). Data were evaluated by univariate analysis of the general linear model and multivariate linear regression analysis. The Kolmogorov–Smirnov test was used to assess the normality of data. Frequency, percentage, median, minimum, and maximum values were used for descriptive statistics. We used the chi‐square test for categorical variables for the two group comparisons and the Student's *t*‐test and Mann–Whitney *U* test for continuous data. A *p* value of < 0.05 was considered statistically significant.

## 3 Results

Two hundred thirty‐three patients who agreed to participate and met the study criteria were included. MARS scores were used to measure drug adherence. Ninety‐seven patients (41.6%) had a MARS score of less than 20 (Group 1). One hundred thirty‐six patients (58.4%) had a MARS score of over 20 (Group 2). Two groups were compared. The median age was 43 (27–84) years. There was no significant difference between the two groups in terms of age, marital status, duration of ED, presence of chronic diseases (diabetes mellitus, hypertension, coronary artery disease), and additional drug usage. When the education level was evaluated, in Group 1, 61 (62.9%) had more than 8 years of education, and in Group 2, 104 (76.5%). There was a significant difference between the two groups (*p* = 0.024). In multivariate analysis, the difference is not significant (*p* = 0.173). There was a significant difference between the two groups regarding monthly income, frequency of medical examination, and smoking habits (*p* < 0.001). In multivariate analysis, *p* values were 0.029, 0.239, and 0.087, respectively. The proportion of patients with a monthly income of more than 30 000 TL in Group 1 was 13 (13.4%), and in Group 2 it was 57 (42%) (*p* < 0.001). In multivariate analysis, the *p*‐value was 0.029. When the frequency of medical examination was examined, the rate of patients coming for control at 3‐month intervals was 7 (7.2%) in Group 1 and 38 (27.9%) in Group 2 (*p* < 0.001). In multivariate analysis, the *p*‐value was 0.239. When smoking habits were compared, the rate of smokers in Group 1 was 44 (45.3%), and in Group 2 it was 32 (23.5%) (*p* < 0.001). In multivariate analysis, the *p*‐value was 0.087. There was also a statistically significant difference in patients who underwent radical pelvic surgery. Nine (9.2%) patients in Group 1 and two (1.4%) patients in Group 2 had undergone radical pelvic surgery (*p* = 0.006). In multivariate analysis, the *p* value was 0.027. All data comparing the sociodemographic and clinical characteristics of the patients with drug adherence are shown in Table 1.

**TABLE 1:** Sociodemographic and clinical characteristics of erectile dysfunction patients according to medication adherence.

	Total (*n* = 233)	Group 1	Group 2	*p* univariate^a^	*p* multivariate^b^
		Low adherence [MARS median score < 20] (*n* = 97; 41.6%)	High adherence [MARS median score ≥ 20] (*n* = 136; 58.4%)		
Age (years), median (min–max)	43 (27–84)	45 (28–84)	42 (27–82)	0.236	0.451
*Marital status*					
Single	87 (37.3%)	39 (40.2%)	48 (35.3%)	0.446	0.493
Married	146 (62.7%)	58 (59.8%)	88 (64.7%)		
*Education level*					
≤ 8 years	68 (29.2%)	36 (37.1%)	32 (23.5%)	**0.024**	0.173
> 8 years	165 (70.8)	61 (62.9%)	104 (76.5%)		
*Monthly income, US Dollars*					
450–700	59 (25.3%)	38 (39.2%)	21 (15.4%)	**< 0.001**	**0.029**
700–1400	104 (44.6%)	46 (47.4%)	58 (42.6%)		
> 1400	70 (30.1%)	13 (13.4%)	57 (42%)		
*Duration of ED*					
≤ 2 years	135 (58%)	55 (56.7%)	80 (58.9%)	0.737	0.644
> 2 years	98 (42%)	42 (43.3%)	56 (41.1%)		
*Frequency of medical examination*					
0–3 months	45 (19.3%)	7 (7.2%)	38 (27.9%)	**< 0.001**	0.239
3–6 months	53 (22.8%)	16 (16.5%)	37 (27.3%)		
6 months–1 year	88 (37.8%)	44 (45.4%)	44 (32.3%)		
Longer than 1 year	47 (20.1%)	30 (30.9%)	17 (12.5%)		
*Diabetes mellitus*					
Present	48 (20.6%)	17 (17.5%)	31 (22.8%)	0.325	0.534
Absent	185 (79.4%)	80 (82.5%)	105 (77.2%)		
*Hypertension*					
Present	62 (26.6%)	26 (26.8%)	36 (26.5%)	0.959	0.608
Absent	171 (73.4%)	71 (73.2%)	100 (73.5%)		
*Additional drug use*					
Present	83 (35.6%)	32 (32.9%)	51 (37.5%)	0.470	0.384
Absent	150 (64.4%)	65 (67.1%)	85 (62.5%)		
*Smoking habit*					
Present	76 (32.6%)	44 (45.3%)	32 (23.5%)	**< 0.001**	0.087
Absent	157 (67.4%)	53 (54.6)	104 (76.5%)		
*Coronary artery disease*					
Present	28 (12.1%)	10 (10.3%)	18 (13.3%)	0.489	0.672
Absent	205 (87.9%)	87 (89.7%)	118 (86.7%)		
*Radical pelvic surgery*					
Present	11 (4.7%)	9 (9.2%)	2 (1.4%)	**0.006**	**0.027**
Absent	222 (95.3)	88 (90.8%)	134 (98.6)		

*Note:* *p* < 0.05 is accepted as significant and shown in bold.

Abbreviations: ED, erectile dysfunction; MARS, Medication Adherence Report Scale.

^a^ Univariate analysis of the general linear model.

^b^ Multivariate linear regression analysis.

In the B‐IPQ scale, the two groups significantly differed in the consequences, personal control, treatment control, understanding concern, and emotional representation (*p* = 0.019, *p* = 0.041, *p* = 0.029, *p* = 0.016, *p* = 0.033, respectively). In multivariate analysis, *p* values were 0.211, 0.045, 0.043, 0.027, and 0.031, respectively. Consequences, understanding concern, and emotional representation subscale scores were higher in Group 1. Group 2 had higher personal control and treatment control subscale scores. BMQ‐specific concerns subscale scores, BMQ‐specific necessity subscale scores, and BMQ‐general harm subscale scores were significantly different (*p* = 0.027, *p* = 0.021, *p* = 0.034, respectively). In multivariate analysis, *p* values were 0.034, 0.029, and 0.022, respectively. The data of these results are shown in detail in Table 2.

**TABLE 2:** Psychological factors of erectile dysfunction patients according to medication adherence.

	Total (*n* = 233)	Group 1	Group 2	*p* univariate^a^	*p* multivariate^b^
		Low adherence [MARS median score < 20] (*n* = 97; 41.6%)	High adherence [MARS median score ≥ 20] (*n* = 136; 58.3%)		
BIPQ					
Consequences	6 (0–9)	7 (0–9)	5 (0–8)	**0.019**	0.211
Timeline	5 (0–10)	6 (1–10)	5 (0–10)	0.224	0.681
Personal control	8 (0–10)	6 (0–9)	9 (0–10)	**0.041**	**0.045**
Treatment control	7 (0–9)	5 (0–9)	8 (0–9)	**0.029**	**0.043**
Identity	4 (0–10)	4 (0–10)	3 (0–9)	0.087	0.194
Understanding, concern	6 (1–9)	7 (1–9)	5 (1–8)	**0.016**	**0.027**
Illness coherence	8 (2–10)	7 (2–9)	8 (2–10)	0.108	0.391
Emotional representation	7 (0–10)	9 (0–10)	6 (0–9)	**0.033**	**0.031**
Total	50 (24–66)	50 (31–66)	49 (24–59)	0.389	0.866
BMQ‐specific					
Concerns	3 (1.2–4)	3.4 (1.4–4)	2.8 (1–3.6)	**0.027**	**0.034**
Necessity	2.8 (1–4.2)	2.4 (1.2–4)	3.2 (1–4.4)	**0.021**	**0.029**
Necessity beliefs (differential)	−0.4 (−2.4–3.8)	−0.8 (−2.8–3.4)	0.4 (−2.4–3.2)	0.122	0.177
BMQ‐general					
Overuse	1.75 (1–4.5)	2 (1.25–4.5)	1.75 (1–4)	0.193	0.266
Harm	2.25 (1.5–4.25)	2.75 (1.5–4.25)	2 (1.5–4)	**0.034**	**0.022**
BMQ‐total score	8.20 (6.2–13.15)	8.50 (6.85–13.15)	8 (6.2–12.75)	**0.015**	0.157

*Note:* Data given as median (min–max); values of *p* < 0.05 were accepted as significant and are shown in bold.

Abbreviations: B‐IPQ, The Brief Illness Perception Questionnaire; BMQ, Beliefs about Medicines Questionnaire; MARS, Medication Adherence Report Scale.

^a^ Univariate analysis of the general linear model.

^b^ Multivariate linear regression analysis.

Drug adherence was compared between different ED levels. IIEF scores were determined before receiving tadalafil 5 mg OaD treatment. The number of patients with severe ED was 38 (39.1%) in Group 1 and 27 (19.8%) in Group 2 (*p* < 0.001). The data are shown in detail in Table 3.

**TABLE 3:** IIEF scores of erectile dysfunction patients according to medication adherence.

	Total (*n* = 233)	Group 1	Group 2	
		Low adherence [MARS median score < 20] (*n* = 97; 41.6%)	High adherence [MARS median score ≥ 20] (*n* = 136; 58.3%)	
26–30 no erectile dysfunction	3 (1.3%)	1 (1.1%)	2 (1.4%)	*p* < **0.001**
22–25 mild erectile dysfunction	40 (17.2%)	10 (10.3%)	30 (22.1%)	
17–21 mild to moderate erectile dysfunction	54 (23.1%)	14 (14.4%)	40 (29.4%)	
11–16 moderate erectile dysfunction	71 (30.5%)	34 (35.1%)	37 (27.2%)	
0–10 severe erectile dysfunction	65 (27.9%)	38 (39.1%)	27 (19.8%)	

*Note:* *p* < 0.05 is accepted as significant and shown in bold.

Abbreviation: IIEF, International Index of Erectile Function.

## 4 Discussion

PDE‐5 inhibitors have been extensively used for many years in the treatment of ED. Among these, tadalafil is particularly notable for its higher bioavailability and longer half‐life (17.5 h) [20, 21]. Tadalafil can be used as a PRN treatment and an OaD regimen. However, studies have demonstrated that OaD use is associated with greater success when considering psychosocial outcomes, such as improved sexual self‐confidence, enhanced spontaneity, and reduced time‐related concerns [22, 23]. However, studies have noted a low rate of treatment continuation. In the Men's Attitudes to Life Events and Sexuality epidemiological study, oral therapy was initiated in 58% of patients with ED, but only 16% of these patients continued the treatment [24]. This finding underscores the critical role of MA in the success of ED treatment. According to our research, our study is the first to evaluate MA in ED using the BPQ, BIP‐Q, and MARS scales.

MA can be defined as the consistent use of a prescribed drug at the recommended dose and frequency without omissions or modifications. A significant difference was observed in education level between patients with low and high MA (*p* = 0.024). However, this difference was not statistically significant after multivariate analysis (*p* = 0.173). The higher adherence observed in patients with greater educational attainment may be attributed to their prior knowledge of maintaining continuity in pharmacological treatment. In another study on the subject, patients diagnosed with Ankylosing Spondylitis and using antirheumatic drugs were evaluated. In the study by Tolu et al. [25], no significant association was found between education level and MA (*p* = 0.990). This observed disparity suggests the potential presence of societal stigma or bias surrounding the use of PDE‐5 inhibitors, such as Tadalafil 5 mg OaD, for the management of ED. Patients' prejudices against Tadalafil 5 mg OaD treatment may decrease with increasing education level. Therefore, it will be critical for the success of the treatment to provide extra information about drug adherence in PDE‐5 treatment for patients with lower education levels by clinicians.

In our study, when the monthly incomes of the patients were examined, it was shown that patients in the high‐income group had higher MA than those in the low‐income group. This difference was found to be statistically significant (*p* < 0.001). A significant difference was also seen after multivariate analysis (*p* = 0.029). In a study performed by Basim et al. [26], levothyroxine medication compliance was evaluated according to monthly income level, and it was observed that monthly income level significantly affected MA (*p* = 0.03). This finding may be associated with patients' reduced ability to afford the medication due to decreased purchasing power. In our country, this issue is particularly pronounced, as PDE‐5 inhibitors are not covered by health insurance for the treatment of ED. This economic barrier should be considered when planning PDE‐5 inhibitor therapy to ensure optimal patient access and adherence.

Group 2 underwent more frequent medical examinations (*p* < 0.001). It may be related to patients' concerns about the use of PDE‐5 inhibitors, such as addiction and side effects. Additionally, this finding may be attributed to some patients' difficulty fully comprehending treatment instructions during a single session. Although a significant difference was observed in the univariate analysis, it was not retained after multivariate analysis (*p* = 0.239), which may be related to the sample size of our study. Nevertheless, we consider the significant difference in the univariate analysis clinically meaningful. Our observations indicate that some patients struggle to understand the pharmacokinetics of the medication, leading to improper dosing and timing of administration. Therefore, to optimize treatment outcomes with PDE‐5 inhibitors, patients must undergo regular evaluations and receive reinforcement of treatment instructions at least every 3 months.

Considering the effects of chronic diseases, habits, and operation histories on drug adherence, a significant difference is observed among patients who have undergone radical pelvic surgery. Drug adherence was significantly lower in these patients than in those who did not undergo surgery (*p* = 0.006, 0.027 in univariate and multivariate analyses, respectively). When asked about the cause of this outcome, some patients cited inadequate therapeutic benefit from Tadalafil 5 mg OaD. It may be due to the limited efficacy of PDE‐5 inhibitors in certain neurogenic and vascular forms of ED resulting from radical pelvic surgery. It may be considered to increase the dosage of tadalafil for daily use in the treatment of patients following radical pelvic surgery. DM, HT, coronary artery disease, and smoking habits were investigated in parallel. Of these, only the smoking habit was shown to affect MA adversely (*p* < 0.001). Vascular damage caused by smoking may have impaired motivation in treatment. However, similar results could be expected in these diseases due to vascular or neurogenic damage. No significant difference was observed in our study after multivariate analysis (*p* = 0.087). Studies involving larger patient numbers will give more precise information on this subject.

The B‐IPQ test is a scale that provides information about the relationship of patients' cognitive and emotional states with the disease. Specific issues related to the person's emotional well‐being, sense of control over the disease and treatment, and level of anxiety can be expected to affect patients' MA. Anxiety may reduce MA due to fear of side effects or misconceptions. A broader meta‐analysis by DiMatteo et al. concluded that anxiety negatively impacts MA across chronic conditions, likely due to avoidance behaviors and difficulty in managing treatment routines [27]. We hypothesize that in patients with anxiety, the urgency to experience the drug's full therapeutic effect may negatively influence MA to tadalafil 5 mg OaD treatment. Managing anxiety and setting realistic expectations may significantly improve adherence and overall treatment outcomes. Our study also showed that misperception and anxiety affect medication compliance. In another study, urate‐lowering therapy given to gout patients was evaluated [28]. It has been shown that the disease perception results obtained using B‐IPQ are directly related to disease activity. After recent research on ED, the organic causes of the disease have also been elucidated. Accordingly, different and more effective treatment options have emerged [29]. However, this time, urologists focused entirely on organic causes in the treatment of ED. Especially in recent years, cognitive and emotional sides began to be ignored. For this reason, in the planning of ED treatment, the cognitive and sensory aspects of the disease should be examined by urologists without neglecting both causes, along with organic causes. In this way, it is thought that the success of medical treatment will increase.

BMQ was used to evaluate patients' beliefs about drugs. Specific concerns about drugs were questioned in the BMQ‐specific concerns subscale. High scores on this scale indicated greater concern about drug use. MA was adversely affected in high‐score patients (*p* = 0.027, multivariate *p* = 0.034). In BMQ‐specific necessity subscale, their thoughts on the necessity of drug use were questioned. High scores indicated a belief in the necessity of the drug. It positively affected drug adherence (*p* = 0.021, multivariate *p* = 0.029). In the BMQ‐general harm subscale, patients' belief in the harmful effects of drugs is determined. High scores indicated patients' strong belief that drugs had a harmful impact. Naturally, it was observed that this situation adversely affected the drug adherence of the patients (*p* = 0.034, multivariate *p* = 0.022). Considering all these, it can be said that there is a prejudice against the continuous use of PDE‐5 inhibitors in some patients. There are numerous unlicensed food supplements and drugs advertised on the internet and social media that are said to treat ED. Patients may also generalize PDE‐5 inhibitors and include them in this group. It would be beneficial to inform patients by healthcare institutions and organizations.

When patients were assessed based on their IIEF scores, MA was lower in the group with moderate and severe ED compared to the other groups (*p* < 0.001). The presence of advanced ED in these patients may be due to severe neurogenic and vascular diseases. Accordingly, tadalafil 5 mg OaD may have been insufficient in the treatment. The patient, who cannot be treated to the extent he wants, may be indifferent to the treatment. In a randomized study by Buvat et al. [30], 770 ED patients were treated with tadalafil 5 mg OaD, tadalafil 10 mg PRN, and sildenafil 50 mg PRN and were randomized into three groups. The period from baseline to discontinuation of treatment was evaluated as treatment adherence. As a result, the lowest treatment adherence was found in the sildenafil 50 mg PRN group. Higher treatment adherence was found in the tadalafil 10 mg PRN group than in the tadalafil 5 mg OaD group. It has been stated that the most important reason for discontinuation of treatment in the tadalafil 5 mg OaD group is the lack of sufficient efficacy for a hard erection. It can be thought that the use of tadalafil PRN in moderate and severe ED patients may provide more benefits to drug adherence. Alternative treatment methods are available for patients who do not benefit from tadalafil 5 mg OaD treatment or do not comply with treatment. Vacuum devices can be shown among these [31]. In addition, ESWT and magnetic stimulation are other options for treating ED as current treatment methods.

Clinical studies have demonstrated that the most frequently reported adverse effects associated with tadalafil 5 mg OaD include headache, dyspepsia, back pain, myalgia, flushing, nasal congestion, and dizziness. These adverse events are typically mild to moderate in severity and tend to manifest within the first month of therapy. The overall incidence has been reported in approximately 5% of patients [32]. In a study investigating the hemodynamic effects of tadalafil, it was observed that the drug significantly reduced the 26‐h mean blood pressure in a cohort of patients with uncontrolled hypertension [33]. This hemodynamic effect has been observed at tadalafil 20 mg PRN, with a low incidence of postural hypotension. Given that tadalafil 5 mg OaD was administered in our study, it is reasonable to expect a substantially attenuated hemodynamic response. The observed reduction in blood pressure may be regarded as a beneficial effect, provided that serious adverse events, such as syncope, are carefully monitored. In our study, postural hypotension was identified during follow‐up in six patients, all of whom were concurrently receiving antihypertensive therapy. The issue was successfully managed by adjusting their antihypertensive regimen. Further studies with shorter follow‐up intervals and controlled designs are warranted to more comprehensively assess the impact of tadalafil‐associated adverse effects on MA.

In the evaluation of MA in patients receiving tadalafil treatment, a 5 mg OaD regimen was selected, as there is no standard regimen for PRN use. Furthermore, evidence supporting the efficacy and safety of tadalafil 20 mg OaD basis remains insufficient [34]. In addition, a study evaluating the 24‐week follow‐up period reported that OaD treatment had a lower incidence of treatment‐emergent side effects compared to PRN use [35].

There are several limitations to our study. First, all the scales utilized were based on self‐reported data, which may introduce bias and affect the accuracy of the results. Furthermore, the sample size in this cross‐sectional study is relatively small, and larger studies are required to draw more definitive conclusions.

## 5 Conclusion

Tadalafil 5 mg OaD can be utilized in treatment with acceptable MA rates. However, factors such as the patient's socioeconomic status, smoking habits, history of radical pelvic surgery, cognitive and emotional conditions, as well as biases toward medications and healthcare providers can significantly influence adherence. Given the lower MA observed in patients with lower socioeconomic status, clinicians should exercise caution when prescribing Tadalafil 5 mg OaD in this population.

## Author Contributions

E.K. conceived the study, and all authors participated in the study design. E.K. and O.K. collected the data. O.K. analyzed the data. E.K. and O.K. drafted the manuscript. All authors commented on the earlier versions of the manuscript. All authors edited the manuscript and approved the final version.

## Disclosure

The authors have nothing to report.

## Ethics Statement

The Ethics Committee of Karamanoğlu Mehmetbey University Faculty of Medicine approved the study protocol. (Date: 23.06.2021. Decision No: 04‐2021/05). All steps of the study were completed in accordance with the Declaration of Helsinki.

## Consent

Written and verbal consent was obtained from all patients. Written informed consent for publication of their clinical details and/or clinical images was obtained from the patient/parent/guardian/relative of the patient. A copy of the consent form is available for review by the Editor of this journal.

## Conflicts of Interest

The authors declare no conflicts of interest.

## Data Availability

The datasets used and/or analyzed during the current study are available from the corresponding author upon reasonable request.
